# Women with Premenstrual Dysphoria Lack the Seemingly Normal Premenstrual Right-Sided Relative Dominance of 5-HTP-Derived Serotonergic Activity in the Dorsolateral Prefrontal Cortices - A Possible Cause of Disabling Mood Symptoms

**DOI:** 10.1371/journal.pone.0159538

**Published:** 2016-09-12

**Authors:** Olle Eriksson, Anders Wall, Ulf Olsson, Ina Marteinsdottir, Maria Holstad, Hans Ågren, Per Hartvig, Bengt Långström, Tord Naessén

**Affiliations:** 1 Department of Women’s and Children’s Health, Obstetrics and Gynaecology, Uppsala University Hospital, Uppsala, Sweden; 2 Department of Surgical Sciences, Unit for Nuclear Medicine and PET, Uppsala University Hospital, Uppsala, Sweden; 3 Unit of Applied Statistics and Mathematics, Swedish University of Agricultural Sciences, Uppsala, Sweden; 4 Department of Clinical and Experimental Medicine, Linköping University, Linköping, Sweden; 5 Department of Neuroscience, Psychiatry Unit, Uppsala University Hospital, Uppsala, Sweden; 6 Institute of Neuroscience and Physiology, University of Gothenburg, Gothenburg, Sweden; 7 Department of Drug design and Pharmacology, University of Copenhagen, Copenhagen, Denmark; 8 Department of Biochemistry and Organic Chemistry, Uppsala University, Uppsala, Sweden; University of Medicine & Dentistry of NJ - New Jersey Medical School, UNITED STATES

## Abstract

**Study Objective:**

To investigate potential quantitative and qualitative differences in brain serotonergic activity between women with Premenstrual Dysphoria (PMD) and asymptomatic controls.

**Background:**

Serotonin-augmenting drugs alleviate premenstrual mood symptoms in the majority of women with PMD while serotonin-depleting diets worsen PMD symptoms, both indicating intrinsic differences in brain serotonergic activity in women with PMD compared to asymptomatic women.

**Methods:**

Positron-emission tomography with the immediate precursor of serotonin, 5-hydroxytryptophan (5-HTP), radiolabelled by ^11^C in the beta-3 position, was performed in the follicular and luteal phases for 12 women with PMD and 8 control women. Brain radioactivity–a proxy for serotonin precursor uptake and synthesis–was measured in 9 regions of interest (ROIs): the right and left sides of the medial prefrontal cortex, dorsolateral prefrontal cortex, putamen and caudate nucleus, and the single “whole brain”.

**Results:**

There were no significant quantitative differences in brain 5-HTP-derived activity between the groups in either of the menstrual phases for any of the 9 ROIs. However, multivariate analysis revealed a significant quantitative and qualitative difference between the groups. Asymptomatic control women showed a premenstrual right sided relative increase in dorsolateral prefrontal cortex 5-HTP derived activity, whereas PMD women displayed the opposite (p = 0.0001). Menstrual phase changes in this asymmetry (premenstrual—follicular) correlated with changes in self ratings of ‘irritability’ for the entire group (r_s_ = -0.595, p = 0.006). The PMD group showed a strong inverse correlation between phase changes (premenstrual—follicular) in plasma levels of estradiol and phase changes in the laterality (dx/sin) of radiotracer activity in the dorsolateral prefrontal ROI (r_s_ = -0.635; 0.027). The control group showed no such correlation.

**Conclusion:**

Absence of increased premenstrual right-sided relative 5-HTP-derived activity of the dorsolateral prefrontal cortices was found to strongly correlate to premenstrual irritability. A causal relationship here seems plausible, and the findings give further support to an underlying frontal brain disturbance in hormonally influenced serotonergic activity in women with PMD. Because of the small number of subjects in the study, these results should be considered preliminary, requiring verification in larger studies.

## Introduction

Premenstrual dysphoric disorder (PMDD) is characterized by the cyclical occurrence of negative mood symptoms in the late luteal phase of the menstrual cycle [1]. The cardinal mood symptoms of PMDD are irritability, depression of mood, fatigue, affective lability and impaired impulse control [2]. These symptoms are elicited by sexual hormones of ovarian origin [3] and appear in the premenstrual phase of the menstrual cycle, disappearing completely during the course of menstruation and absent in the follicular phase [1]. All of these symptoms can be alleviated by drugs that increase serotonin signalling in the brain [4]. This was first shown by Eriksson et al. [5] and, to date, selective serotonin-reuptake inhibitors (SSRIs) are universally accepted as the most effective medications for PMDD [6, 7]. SSRI treatment provides symptom relief or symptom cure for the majority of affected women. Diets [8] that decrease serotonin signalling have the opposite effect–worsening PMDD symptoms. Together, these results indicate intrinsic differences in brain serotonergic activity between women with PMDD and asymptomatic women.

The serotonin system of the brain is phylogenetically very old and has been remarkably preserved during evolution, implying that it has an important function [9]. The serotonin system has a dual role in transmission and neuromodulation, regulating the effects of other transmitters in the brain and crucial for the regulation of mood, aggression, sexual function, appetite and feeding, thermoregulation, and sleep and wakefulness [10]. A large number of disorders have been linked to serotonergic dysfunction, including depression, anxiety, social phobia, obsessive-compulsive and panic disorders, among others [10].

One important aspect of serotonergic dysfunction seems to be that of impaired impulse control [11] probably associated with an inability to disregard noise from signals [12]. To date twenty different serotonin receptors have been cloned, and fourteen of these are present in the human brain; thirteen are G-protein coupled and one is ion-channel coupled. In the human cortex, and especially in the frontal cortex, the two most important serotonin receptors are the 5-hydroxytryptamine (5HT)2a receptor, which is the most abundant serotonin receptor in this location and exerts excitatory action, and the 5HT1A receptor which is somewhat less abundant and exerts inhibitory action [13]. Both receptors modulate the activity of the main cortical neural constituents: the activating glutamatergic pyramidal cells and the inhibitory GABAergic interneurons [13]. Ovarian hormones can increase the number of 5HT2a receptors [14] but have little or no effect on the expression of 5HT1A receptors [15].

Neuroimaging studies have revealed ovarian steroid modulation of brain activity in regions and circuits relevant to the symptoms of PMDD, including the functions of the prefrontal cortex, the reward systems, and the stress circuitry [16–20]. The dorsolateral prefrontal cortex seems to play a crucial role in controlling impulsive behaviour [21] and reactive aggression [22]. Converging evidence from animal and human neuropsychological and neurological studies and human neuropsychiatric studies suggests that aggressive behaviour is mainly associated with a functional disturbance in the prefrontal cortex [23–25]. It has therefore been hypothesized that the prefrontal cortex modulates behavioural control by providing inhibitory inputs to subcortical circuits (e.g. hypothalamus and amygdala) that might otherwise induce aggression [23, 26]. This would probably also be true for control of impulsive behaviour and irritability outbursts.

In a previous pharmacological treatment study of women with PMD, we showed that the partial 5-HT1A receptor agonist buspirone reduced symptom irritability significantly better than placebo [27]. It has been suggested that the efficacy of serotonin-reuptake drugs (SRIs) and SSRIs in the treatment of PMDD is largely due to an anti-irritability effect [4]. This is in line with animal experiments which have suggested that the anti-aggressive effect of serotonin is partially mediated by 5-HT1A receptors [28]. In this study we used positron-emission tomography (PET) with 5-hydroxytryptophan (5-HTP), the immediate precursor of serotonin, radioactively labelled with an ^11^C isotope in the beta-3 position, as tracer substance to investigate potential quantitative and qualitative differences in brain serotonergic activity between women with PMD–an unorthodox equivalent of PMDD–and asymptomatic controls.

The tracer molecule, [^11^C]5-HTP, given as an intravenous bolus-dose injection, is known to pass the blood-brain barrier in exactly the same way as the non-radioactive 5-HTP molecule, and is taken up by active transportation, independent of blood flow, into aminergic neurons in which the enzyme aromatic amino acid decarboxylase (AADC; also named dopamine decarboxylase or DOPAcarboxylase) decarboxylates the precursor molecule to form ^11^C-serotonin [29, 30]. The ^11^C isotope in the beta-3 position is thus retained in the ^11^C-serotonin molecule [30–33]. Serotonin is not able to pass the blood-brain barrier. The positron-emission radioactivity then registered is thus derived from [^11^C]5-HTP taken up by aminergic neurons, from ^11^C-serotonin formed and stored in those neurons, and from, to a lesser extent, ^11^C-serotonin released into the synaptic space, receptor-bound ^11^C-serotonin and ^11^C-serotonin that has been reuptaken from the synaptic space [31].

All AADC-containing aminergic neurons can take up [^11^C]5-HTP and form ^11^C-serotonin [34]. Thus, the regional radioactivity reveals the distribution and relative concentration of the enzyme AADC [31]. In the human brain, the highest concentrations of AADC are seen in the striatum, with maximum concentrations in the putamen, followed by the caudate nucleus [34]. However, most of the AADC-containing neurons in the striatum are dopaminergic, although serotonergic neurons are also abundant [35]. Concentrations of AADC are low in the frontal cortex (about 10% of those in the striatum) [34], and most AADC-containing neurons in this region are serotonergic; only a small fraction are dopaminergic [36].

Accordingly, this study was designed to investigate potential differences in brain serotonergic activity–quantitative as well as qualitative–using PET and the tracer substance [^11^C]5-HTP, between a group of women with diagnosed severe PMD and a symptom-free control group.

## Methods

### Participants

Twenty women were recruited for the study; twelve fulfilled the criteria for PMD and eight had no premenstrual mood symptoms.

### Subjects with PMD

Of the twelve women fulfilling the PMD criteria, five were recruited through our gynaecological clinic and seven had participated in an earlier treatment trial [27]. Radiotracer data from the first eight subjects scanned were employed for a preliminary correlational report [37].

The inclusion criteria for the PMD subjects were: fulfilment of criteria A-C of Premenstrual Dysphoric Disorder (PMDD) as described in DSM IV [1] and fulfilment of criterion D by showing cyclicity of the core symptoms”irritability” and/or”depressed mood” in two of three visual analogue scale (VAS)-rated cycles. The inclusion criteria were thus a slightly modified version of the PMDD criteria in DSM-IV [1]. The initial VAS instrument (0–100 mm) included the symptoms "irritability", "depressed mood", "tension", "affective lability", "food craving", "breast tenderness" and "a sense of bloating". A mean rating for the five days preceding menstrual onset should be at least 100% higher than that for days 6–10 from menstrual onset, with a minimal luteal phase mean of 30 mm on the VAS scale. Additional inclusion criteria were: fertile age (18–45 years); regular ovulatory menstrual cycles of 22–35 days [verified through daily menstrual cycle VAS-ratings, early follicular phase serum follicle-stimulating hormone (FSH) values in the normal fertile range, and menstrual cycle day 21 serum progesterone values indicative of corpus luteum formation]; stated completion of childbirth; effective non-hormonal contraception; and a normal gynaecological examination within the last year. Right-handedness was also required. Exclusion criteria were: pregnancy or lactation; bleeding irregularity; history of any major psychiatric disorder other than depression, including former or present drug abuse; ongoing depression or a depressive episode within the past two years; ongoing somatic illness; ongoing medication; and left-handedness. One of the study psychiatrists (I.M. or M.H.) also carried out a Structured Clinical Interview [38, 39] of each subject, to rule out psychiatric axis I or II disorders [1].

### Symptom-free control subjects

Eight symptom-free control women, age matched to the respective cases within ± 2 years, were recruited from centres for cervical Papanicolau (Pap) smear screening in Uppsala, Sweden. The automated triennial screening programme has a total coverage of about 70%, ‘opportunistic’ screening included. Midwives engaged in Pap smear screening handed out information sheets about the study to women of the ages required for controls. Women interested in participating in the study underwent a structured telephone interview and a clinical evaluation identical to that for the subjects with PMD. The control women also rated daily symptoms over two menstrual cycles, using a VAS instrument identical to that used by the PMD group. Inclusion criteria for controls were: self-stated absence of premenstrual mood symptoms and associated physical symptoms. Exclusion criteria were: VAS-rated symptom cyclicity indicating PMD. Other inclusion and exclusion criteria were the same as those for the PMD group.

All subjects gave their informed consent to participate in the study, which was approved by the Human Ethics Committee of the Faculty of Medicine, Uppsala University, and by the Radiation Hazards Committee of Uppsala University Hospital. Informed consent was given verbally, according to the current Human Ethics Committee standards, and was documented in the University Hospital record for each study participant.

### Symptom evaluation

During the course of the study, all subjects performed daily prospective self-ratings for four negative and four positive mood variables and six somatic symptoms associated with PMD, using a VAS instrument (0–100 mm) developed by Bäckström et al. and slightly modified from their first description [40]. The assessed variables were: irritability, depressed mood, fatigue, tension, happiness, energy, relaxation, friendliness, headache, bloating, breast tenderness, pelvic pain, craving for sweets and sexual desire. The subjects were instructed to mark total absence of a symptom as 0 mm on the VAS scale and the most intense form of the variable ever experienced by the individual as 100 mm.

### Imaging

The study was carried out in a stepwise fashion and the subjects were therefore subdived into three cohorts for PET registration. Cohort I consisted of eight subjects with PMD who had PET during 1996–1999, Cohort II comprised six control and four PMD women who had PET during 2004–2005, and Cohort III comprised the two remaining control women who had PET during 2011–2012. PET scanners and software were replaced during this period. The protocols for scanning and evaluation were thoroughly adapted to minimize the consequences of these replacements.

### PET scanners

The dedicated brain scanner GEMS PC2048-15B (General Electric Medical Systems, Milwaukee, WI, USA), which was used for Cohort I, had an axial field of view of 15 cm and 15 image planes spaced 6.5 mm apart. The whole body ECAT EXACT HR+ scanner (Siemens CTI), which was used for Cohorts II and III, had an axial field of view of 15.5 cm and 63 slices spaced 2.46 mm apart. The in-plane resolution, after application of a 6 mm post-reconstruction filter, was 8.1 mm for both scanners.

### PET scanning protocol

PET data were collected using identical acquisition protocols: ^15^O-H_2_O scans were recorded in 15 frames of 10 s and [^11^C]5-HTP scans were recorded in five frames of 60 s, five frames of 120 s, five frames of 180 s and six frames of 300 s. A transmission scan preceding tracer injection corrected for attenuation. The ^15^O-H_2_O and [^11^C]5-HTP scans were done in consecutive order. PET images were acquired in 2D mode for Cohorts I and II and in 3D mode for Cohort III.

The goal tracer doses for Cohort I were 15 MBq and 6 MBq per kg bodyweight for ^15^O-H_2_O and [^11^C]5-HTP. The goal tracer doses for Cohort II (20 MBq of ^15^O-H_2_O and 10 MBq of [^11^C]5-HTP per kg bodyweight) were adjusted to compensate for the lower sensitivity of the ECAT EXACT HR+ whole body scanner. The maximum tracer dose was limited to 1600 MBq for ^15^O and 800 MBq for ^11^C. Because Cohort III was registered in 3D mode, the goal tracer doses were reduced (10 MBq and 5 MBq per kg bodyweight for ^15^O-H_2_O and [^11^C]5-HTP, respectively).

All subjects had two PET measurements: in the mid follicular (postmenstrual) and late luteal (premenstrual) phases of the menstrual cycle. To avoid order bias, it was originally planned that half of the subjects in each group would be registered in a ‘reversed’ menstrual cycle, and this was done for Cohort I. However, over time, it became increasingly clear that this introduced unphysiological ‘split cycles’, and this concept was abandoned.

However, for practical reasons, three more subjects had to be registered in a ‘reversed’ menstrual cycle, two from the PMD group and one from the control group.

PET was carried out by 9 a.m., in the fasting state, in the standardized supine position and with standardized afferent inputs as far as light, sound and temperature were concerned. Since it was important that the subjects did not fall asleep, a standardized, recorded tape/CD played melodious, rhythmic but”neutral, cool and uncontroversial” jazz music during the PET scans [41].

### Post-imaging processing

All the images for each subject were lined up, as described by Andersson [42]. First, all [^11^C]5-HTP images were examined and corrected for within-scan movement, and summed images were made from the follicular and luteal phase scans for each subject. Since the ^15^O-H_2_O and [^11^C]5-HTP scans were done consecutively with the subjects remaining in the scanner, no inter-scan realignment for ^15^O-H_2_O and [^11^C]5-HTP was needed in most cases, but it was done if the subject moved. Thereafter, the ^15^O-H_2_O and [^11^C]5-HTP images acquired in the follicular phase were lined up with the ^15^O-H_2_O and [^11^C]5-HTP images acquired in the luteal phase. This procedure allowed one set of regions of interest (ROIs) to be delineated and used per subject.

**Fig 1:**
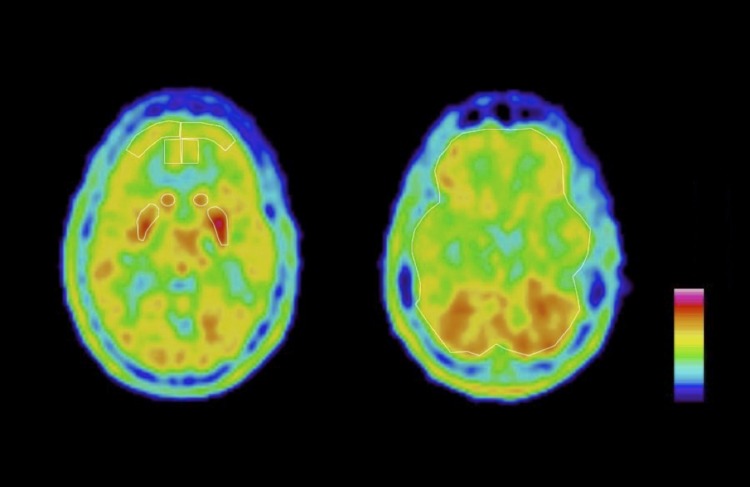
PET-images of the 9 brain regions of interest (ROIs). The nine brain ROIs depicted on [^11^C]5-HTP summation images from one PMD subject, showing the eight frontal ROIs (left picture) and one ‘whole brain’ ROI (right picture).

The ROIs were drawn on the ^15^O-H_2_O summed images, which allowed identification of the anatomical landmarks, and were then copied to the [^11^C]5-HTP summed images to optimize the anatomical precision. These ROIs were then checked again for accuracy in the [^11^C]5-HTP summed images. Nine ROIs were delineated: dorsolateral prefrontal cortex, medial prefrontal cortex, putamen and caudate nucleus, each on the right and left side; and a single whole brain ROI (see Fig 1). The ROIs delineated in consecutive slices were linked to volumes of interest (VOIs) as previously described [37]. The Image Display and Directory computer program (Scanditronix/General Electric, Uppsala, Sweden) was used to delineate ROIs for Cohorts I and II, and the VOIAGER 2.0.5 program (GE Healthcare 2009) was used for Cohort III. The dorsolateral, prefrontal and medial prefrontal cortex ROIs were drawn for Cohorts I and II with the assistance of an automated cortex-ROI algorithm that allowed delineation of cortex ROIs with a fixed width of 10 mm. The ROIs for Cohort III were delineated manually with the same cortex width. The eight forebrain regions were chosen on the basis of their known serotonergic innervation and because of their documented involvement in affective disorders [43–45], and the whole brain ROI, just below the level of the basal ganglia, was chosen as a potential reference region but was never used as such. The ROI depiction method was adapted to 30 slices spaced at 4.9 mm intervals for cohort II, since the Scanditronix/General Electric program was limited to 30 image slices. For that reason the images acquired for cohort II were resampled to 30 slices. For cohort III, the method was adapted to 63 slices spaced at 2.46 mm intervals to cover a similar axial field with the VOIs regardless of the scanner or software used.

**Fig 2:**
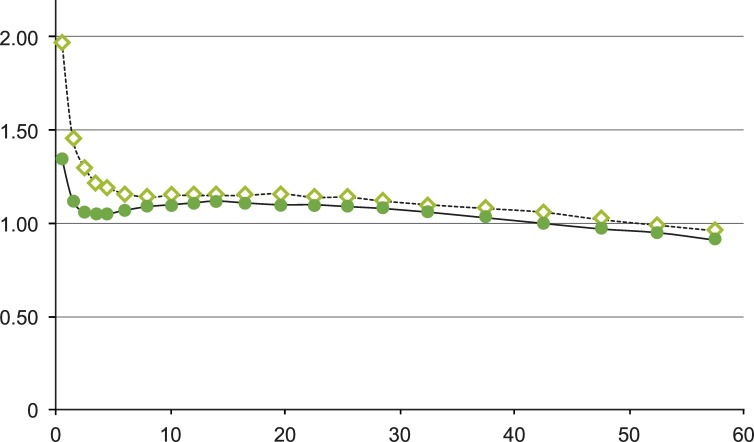
Standardized uptake value (SUV) curves after [^11^C]5-HTP injection. SUV curves of radioactivity in the ‘whole brain’ ROI of a PMD subject after intravenous [^11^C]5-HTP injection in the follicular (dotted line with diamonds) and premenstrual (continuous line with dots) phases of the menstrual cycle.

The regional and dynamic radioactivity trapped in the brain was measured as a dimensionless standardized uptake value (SUV), estimated as the radioactivity measured by the scanner divided by the given tracer dose per kg body weight. Fig 2 shows the SUV curves of radioactivity in the "whole brain" ROI after injection of [^11^C]5-HTP. As stated above, radioactivity was corrected for physical decay from the time of injection. The total radiotracer accumulation in an ROI was calculated as the area under the standardized uptake concentration-time curve (AUC) from 9 to 60 minutes after radiotracer injection (frames 8–21). AUC values were calculated for each ROI for the two menstrual cycle phases in every subject. Because two different PET cameras were used in this study, SUV values calculated from the whole body ECAT EXACT HR+ scanner (Siemens CTI), used for Cohorts II and III, were multiplied by the correction factor 1.19 to correlate with SUV values calculated from the dedicated brain scanner GEMS PC2048-15B (General Electric Medical Systems, Milwaukee, WI, USA), used for Cohort I. This correction factor was established earlier through rigorous research work by the Uppsala University PET Center, with the use of multiple phantoms and radiotracers.

### Statistical analysis

The data consisted of brain activity measurements in the left and right sides of four brain regions during two phases, for patients and controls.

Linear mixed models [46, 47] were used to find whether brain 5-HTP-derived activity and laterality were related to menstrual phase and/or to study group. The models included patients as a random factor and used patient category and phase, and the interaction between these, as fixed factors. Similar mixed models were later used for other variables.

Stepwise discriminant analysis [48] was then used to identify combinations of variables related to patient category.

Post-hoc tests were adjusted for multiplicity using Tukey’s method [49].

All analyses were performed using SAS [50] software. The level of significance was p<0.05, except in the stepwise analysis where p<0.15 was used.

## Results

### Subject characteristics

PMD and control subjects did not differ significantly in age, height, number of pregnancies, number of children born, menstrual cycle length, duration of menstrual bleeding or MADRS score at study entry (Table 1). However, control subjects were significantly heavier and had a higher BMI. All study participants were native Swedish women of Western European origin. The PMD group reported a mean age at onset of PMD symptoms of 25.8 ± 6.6 years, a mean 13.1 ± 7.4 years of experiencing cyclic symptoms, and a mean of 7.9 ± 2.2 premenstrual symptoms over 9.3 ± 3.3 days per menstrual cycle (S1 File).

**Table 1:** Subject characteristics.

Variable	PMD	Controls	p-value
Age (years)	38.9 ± 3.6	38.2 ± 4.8	0.72
Height (cm)	166.0 ± 3.9	170.3 ± 6.7	0.12
Weight (kg)	60.6 ± 6.3	69.6 ± 4.8	0.001
BMI (kg/m²)	22.0 ± 1.8	24.1 ± 2.0	0.03
Number of pregnancies	2.9 ± 1.9	3.6 ± 1.3	0.24
Number of children born	1.8 ± 1.1	2.9 ± 0.8	0.05
Menstrual cycle length (days)	28.3 ± 1.9	27.0 ± 2.0	0.17
Duration of menstrual bleeding (days)	5.7 ± 0.8	5.1 ± 1.0	0.13
MADRS score	1.6 ± 1.6	1.6 ± 1.4	0.96

Plasma estradiol and progesterone levels measured at the time of the PET scans did not differ significantly between the groups in either menstrual phase (Table 2). There were no significant differences in plasma estradiol levels between the phases for either group. The highly significant differences in plasma progesterone levels between the follicular and premenstrual phases were equally strong for both groups (p < 0.0001). The timing of the two PET scans did not differ significantly between the groups with respect to day of the menstrual phase, and was as similar as practically possible. One PMD subject and one control subject were 45 years old by the time they received PET scans. Both of these women were in late reproductive stage -3b according to the 2011 STRAW + 10 reviewed criteria [51].

**Table 2:** Group characteristics at PET registration.

Variable	PMD	Controls	p-value
Day of follicular phase PET	8.9 ± 2.3	10.1 ± 1.5	0.16
Estradiol level at follicular phase PET (pmol/L)	440.4 ± 426.1	610.0 ± 850.9	0.64
Progesterone level at follicular phase PET (nmol/L)	2.1 ± 1.9	1.5 ± 0.9	0.31
Day of luteal phase PET	-3.7 ± 2.4	-4.1 ± 2.9	0.78
Estradiol level at luteal phase PET (pmol/L)	310.6 ± 212.1	416.3 ± 295.8	0.28
Progesterone level at luteal phase PET (nmol/L)	30.0 ± 29.5	20.6 ± 12.9	0.73
Both PET registrations in same cycle	6	7	0.16
PET registrations in two cycles	6	1	0.16

### PET results

Mixed-model analysis showed no significant differences in the [^11^C]5-HTP-derived brain radioactivity AUCs between the follicular and luteal phases for any of the 9 ROIs in either of the groups, or between the groups.

When stepwise discriminant analysis was used to examine differences between the menstrual phases in the AUCs of all the ROIs, the right dorsolateral prefrontal cortex and left dorsolateral prefrontal cortex ROIs were selected for further investigation (Wilk’s lambda: p = 0.0008). Since the two selected ROIs belonged to the same functional brain structure, it appeared that the relationship between the sides [*right*: *dexter (dx)* and *left*: *sinister (sin)*] might be of interest, in particular for the dorsolateral prefrontal cortex ROI. Therefore, the AUC ratio (dx/sin) was calculated for each ROI for each menstrual cycle phase. These ratios were analyzed in a linear mixed model as outlined above. The results are summarized in Table 3.

**Table 3:** Outcome of linear mixed-model analysis.

Brain region of interest	Category	Phase	Category*phase
Caudate nucleus	0.12	0.77	0.11
Putamen	0.83	0.13	0.98
Dorsolateral prefrontal cortex	0.30	< 0.004	< 0.0001
Medial frontal cortex	0.67	0.21	0.04

P-values for testing the radiotracer activity ratio (right/left) for the four frontal brain areas of interest.

The * symbol indicating the category by phase interaction.

**Fig 3:**
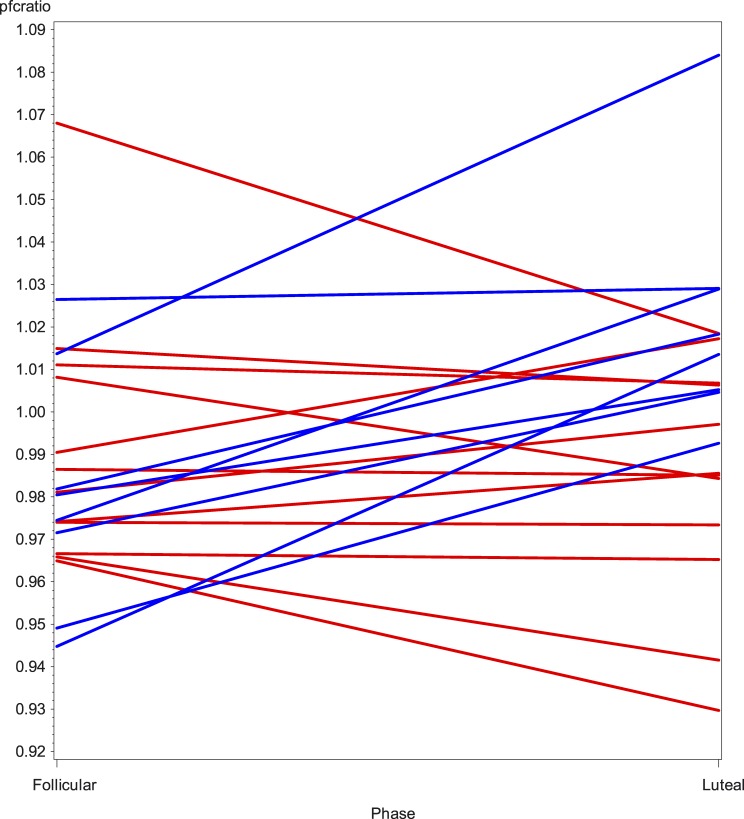
Menstrual phase changes in dorsolateral prefrontal cortex [^11^C]5-HTP-derived radiotracer activity ratios (dx/sin). Plot of the menstrual phase changes in individual values of right-to-left dorsolateral prefrontal cortex ratios of [^11^C]5-HTP-derived radioactivity, subdivided by study group: controls (blue), PMD subjects (red).

A highly significant category*phase interaction was found for the dorsolateral prefrontal cortex AUC ratio (p = 0.0001). To illustrate this, individual values of the dorsolateral prefrontal cortex dx/sin AUC ratio (by menstrual phase, subdivided by study group) are shown in Fig 3.

The general pattern indicated that the dx/sin AUC ratio tended to decrease in women with PMD from the follicular to the premenstrual phase, while the opposite was true for the control women.

Thus, multivariate analysis revealed a highly significant menstrual phase difference in the laterality of tracer-derived radioactivity in the dorsolateral prefrontal cortex between the groups (p = 0.0001).

The mean dx/sin ratio of activity in the dorsolateral prefrontal cortices was 1.02 for controls and 0.98 for women with PMD in the premenstrual phase and 0.98 and 0.99, respectively, in the follicular phase (difference not statistically significant for either phase).

Right-sided dorsolateral prefrontal cortex serotonin-precursor-related radioactivity increased relative to that on the left side in all eight control women during the premenstrual phase. In eight of the twelve women with PMD, radioactivity in the right dorsolateral prefrontal cortex decreased relative to that in the left side during the premenstrual phase. Of the remaining four women, there was almost no difference between sides in one, and there was a slight right-sided increase relative to the left in three.

### Correlations between changes in brain radiotracer activity with menstrual phase and changes in hormone levels

**Fig 4:**
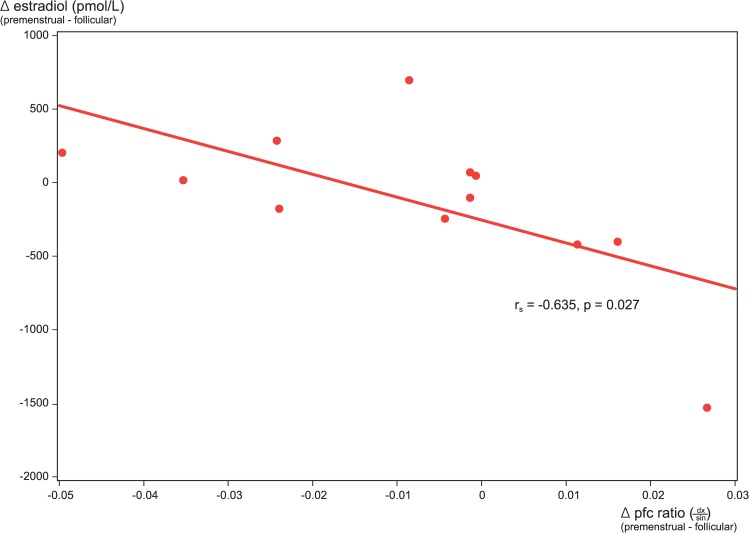
Correlation of menstrual phase changes in plasma estradiol with phase changes in dorsolateral prefrontal cortex [^11^C]5-HTP-derived radioactivity ratios (dx/sin). Plot of menstrual phase changes (premenstrual—follicular) in plasma levels of estradiol, and phase changes (premenstrual—follicular) in the laterality (dx/sin) of radiotracer activity in the dorsolateral prefrontal cortex for the PMD group.

There was a strong inverse correlation between phase changes (premenstrual—follicular) in plasma levels of estradiol and phase changes in the laterality (dx/sin) of radiotracer activity in the dorsolateral prefrontal cortex for the PMD group (r_s_ = -0.635; 0.027), shown in Fig 4.

The corresponding correlation between phase changes in plasma levels of progesterone and phase changes in radiotracer activity laterality, however, was not statistically significant (r_s_ = -0.566; 0.055). There were no correlations between phase changes in plasma levels of estradiol or progesterone and phase changes in the laterality of radiotracer activity in any of the ROIs for the control group.

### Correlations between menstrual phase changes in brain radiotracer activity and changes in VAS-rated symptom scores

**Fig 5:**
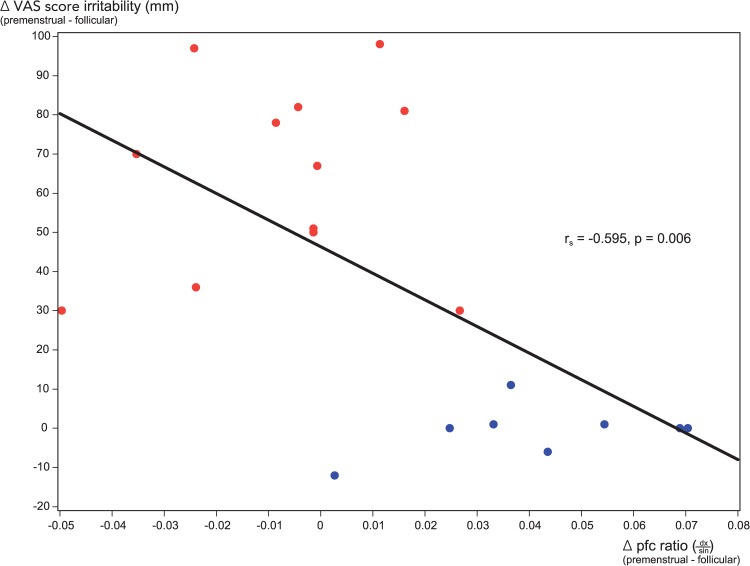
Correlation of menstrual phase changes in VAS scores for the symptom ‘irritability’ with phase changes in dorsolateral prefrontal cortex [^11^C]5-HTP-derived radioactivity ratios (dx/sin). Plot of menstrual phase changes (premenstrual—follicular) in individual VAS ratings for the symptom ‘irritability’, and phase changes (premenstrual—follicular) in the laterality (dx/sin) of radiotracer activity for the entire study group. PMD subjects: red dots; controls: blue dots.

**Fig 6:**
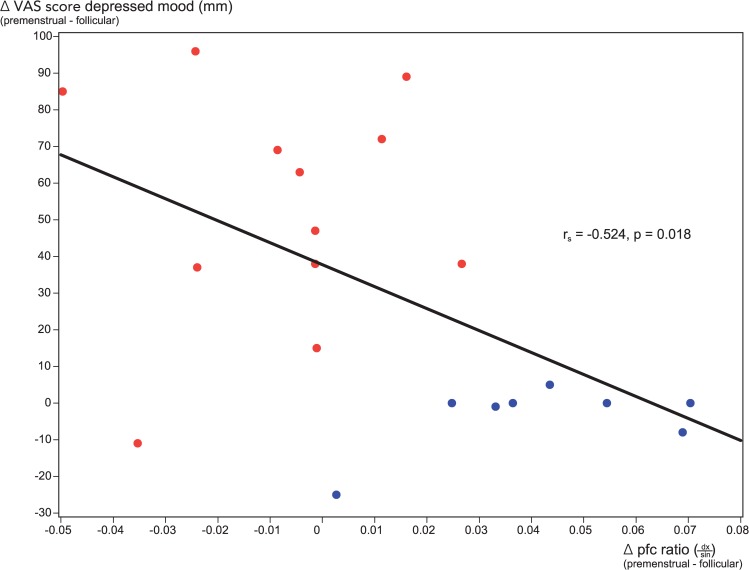
Correlation of menstrual phase changes in VAS scores for the symptom ‘depressed mood’ with phase changes in dorsolateral prefrontal cortex [^11^C]5-HTP-derived radioactivity ratios (dx/sin). Plot of menstrual phase changes (premenstrual—follicular) in individual VAS ratings for the symptom ‘depressed mood’, and phase changes (premenstrual—follicular) in the laterality (dx/sin) of radiotracer activity for the entire study group. PMD subjects: red dots; controls: blue dots.

For the entire study group, menstrual phase changes (premenstrual—follicular) in the laterality (dx/sin) of radiotracer activity in the dorsolateral prefrontal cortex were correlated with changes in VAS symptom scores for 9 of the 14 variables evaluated daily. The strongest negative correlation was seen for the cardinal PMD mood symptom ‘irritability’ (r_s_ = -0.595, p = 0.006), shown in Fig 5, followed by ‘depressed mood’ (r_s_ = -0.524, p = 0.018), shown in Fig 6, ‘pelvic pain’ (r_s_ = -0.489, p = 0.029), ‘fatigue’ (r_s_ = -0.458, p = 0.042), ‘tension’ (r_s_ = -0.451, p = 0.046) and ‘craving for sweets’ (r_s_ = -0.445, p = 0.0495). Positive correlations were seen for ‘happiness’ (r_s_ = 0.574, p = 0.008), ‘friendliness’ (r_s_ = 0.568, p = 0.009) and ‘energy’ (r_s_ = 0.524, p = 0.018). There was no correlation with ‘relaxation’, ‘breast tenderness’, ‘bloating’, ‘headache’ or ‘sexual desire’.

## Discussion

The main finding of this study was the clear and obvious difference in premenstrual right-to-left-sided asymmetry in dorsolateral prefrontal cortex [^11^C]5-HTP-derived brain activity between asymptomatic control women and women with PMD. Among these right-handed women, there was a consistent increase in premenstrual [^11^C]5-HTP-derived brain activity in the right dorsolateral prefrontal cortex relative to the left side in the asymptomatic group, and a relative decrease in [^11^C]5-HTP-derived brain activity in the right dorsolateral prefrontal cortex in the PMD group. The pattern in the control women thus corresponded to the absence of premenstrual mood symptoms and the reverse pattern in women with PMD corresponded to the existence of premenstrual mood symptoms. The findings are correlational, but appear to indicate a causal mechanism.

There was also a strong inverse correlation between menstrual phase changes in plasma levels of estradiol and changes in the dx/sin asymmetry of dorsolateral prefrontal cortex [^11^C]5-HTP-derived activity in women with PMD. Thus, higher levels of plasma estradiol in the premenstrual phase were correlated with lower relative dx/sin dorsolateral prefrontal cortex [^11^C]5-HTP-derived activity ratios and worse mood symptoms. There were no correlations between plasma levels of estrogen and changes in dx/sin dorsolateral prefrontal cortex asymmetry of [^11^C]5-HTP-derived activity in the control women. Thus, this study demonstrated a group difference in the dorsolateral prefrontal cortex response to plasma estradiol levels. Our group has previously reported a difference in the hypothalamus/pituitary response to plasma estradiol levels between PMD and control women [52]. A mood symptom-provoking effect of estradiol in women with PMDD, not displayed in healthy control women, has also been convincingly shown by Schmidt et al. [3].

The brain area in which we find these group differences, the dorsolateral prefrontal cortex or Brodmann areas 9 and 46, is known to have reciprocal connections with other cortical and subcortical regions (e.g. the ventromedial and orbitofrontal prefrontal cortex, parietal cortex, basal ganglia, premotor cortex, supplementary motor area, cingulate cortex, and thalamus) and is known for its role in modulating executive cognitive functions associated with goal-directed behaviour, including attention, self-regulation, planning, inhibition, and control of impulsive behaviour [53]. The serotonin system of the brain has a crucial role in this delicate regulation, where cognitive and emotional information is integrated to form concepts of action, planning of complex cognitive and affective functions and programs, the execution of actions and working memory functions, and the expression of mood [54]. The serotonin innervation of the prefrontal cortex emanates from the median and dorsal raphe nuclei of the brain stem, and the two most important serotonin receptors in this cortex are, as already mentioned, the abundant 5HT2A and the somewhat less abundant 5HT1A receptors. The 5HT2A serotonin receptors are excitatory, expressed in both pyramidal neurons and interneurons, and involved in prefrontal cortex activation [55]. The 5HT1A receptors are inhibitory [56]. These are also expressed in both pyramidal neurons and interneurons, and there appears to be a high degree of co-expression of 5HT2A and 5HT1A receptors in prefrontal cortex neurons [57]. In a seminal rodent experiment, Sumner and Fink (1995) were the first to show that a single dose of estradiol was capable of inducing a significant increase in the density of 5HT2a receptors in the forebrains of female rats [58]. Later studies showed that estradiol priming followed by combined estradiol and progesterone treatment induced widespread increases in 5HT2a receptor binding potential in healthy postmenopausal women [59, 60]. In a PET study of ten right-handed postmenopausal women, Kugaya et al. (2003) showed that ten weeks of transdermal estradiol treatment with hormonal levels in the fertile follicular phase range induced a significant increase in 5HT2a receptor binding, most pronounced in the right frontal cortex [14]. They recorded a 36.5% increase in receptor binding compared to the basal state. Early preclinical studies of the prefrontal cortex identified a very strong 5-HT2a receptor-induced increase in spontaneous glutamate-mediated excitatory synaptic activity [61]. Modern research has stressed that 5-HT2a and 5-HT1A receptors regulate how pyramidal neurones encode excitatory inputs into action potential firing in a cooperative manner [13].

In 1970, Sugerman et al. reported quantitative EEG changes during the human menstrual cycle and suggested that subjective feelings of premenstrual tension appeared to be accompanied by signs of cortical arousal [62]. Since then, a number of studies have shown a premenstrual increase in the right frontal brain EEG relative to the left frontal brain, in parallel with premenstrual symptomatology [63–65]. This is in line with studies showing that, to a great extent, negative emotions are lateralized to the right hemisphere of the brain [66, 67].

In an advanced design study [17] in which parallel PMDD and control groups were scanned during working memory provocations using ^15^O-H_2_O PET and functional magnetic resonance imaging (fMRI), Baller et al. measured regional cerebral blood flow and blood-level-dependent fMRI signals, respectively. PMDD patients had greater prefrontal activation than controls, especially in the dorsolateral prefrontal cortex. This increase in dorsolateral prefrontal cortex activation correlated with the degree of disability, the age at symptom onset, the duration of PMDD, and the menstrual cycle changes in PMDD symptoms. The results implied that dorsolateral prefrontal cortex dysfunction constituted a risk for PMDD, and a ‘trait-like’ predisposition for this hormonally triggered disorder. Their findings give direct support to the findings of this study.

Thus, several studies have shown increased frontal cortex activation, especially in the right frontal cortex, in parallel with premenstrual phase mood symptoms. Increased right-sided prefrontal cortex activity has also been described in patients with major depression [68], anxiety [68], and social phobia [69], conditions which women with PMD have a well-documented increased risk of developing [70].

The lower premenstrual right dorsolateral prefrontal cortex serotonergic activity (relative to left-sided activity) in women with PMD demonstrated in this study was related in time to the cardinal symptom irritability, which implies subnormal serotonergic suppression of intrinsic right prefrontal cortex activity in the premenstrual phase of the menstrual cycle in women with PMD.

This proposed subnormal serotonergic dampening of right prefrontal cortex activity in PMD in the premenstrual phase might possibly be due to reduced activation of cortical 5-HT1A receptors.

In a small PET study of 5-HT1A receptor binding, Jovanovic et al. demonstrated a significantly smaller increase in binding potential from the follicular to the luteal phase in women with PMDD than in control women [71]. This implied serotonergic dysregulation in women with PMDD. Raphe 5-HT1A receptors, which they studied, are presynaptically located and considered excitatory in action, whereas cortical 5-HT1A receptors are postsynaptic and considered inhibitory [72].

In a more recent study, Bismark et al. looked at isoforms of the 5-HT1A receptor and found that variations in the HTR1a gene were related to trait EEG asymmetry, and that subjects with homozygous HTR1a risk alleles had significantly higher relative right frontal activity in dorsolateral frontal areas which, they inferred, may ultimately be indicative of risk of pathology [73].

Extensive research on the effects of 5-HTP/serotonin on the prefrontal cortex has concluded that the net effect is overwhelmingly inhibitory [72]. Given this fact, the main finding in our study of an absent premenstrual relative serotonergic increment in right dorsolateral prefrontal cortex activity in women with PMD is completely in line with the findings of increased right prefrontal cortex EEG activity in women with premenstrual mood symptoms [65]. It is also in line with the findings of Baller et al. of higher dorsolateral prefrontal cortex activation in PMDD patients than in controls [17]. However, Baller et al. did not report any difference in the laterality of the increased activation, which was seen in the PMDD patients in all of three different experimental hormonal settings.

There are some obvious methodological weaknesses in our study. The most fundamental is the small size of the study samples. This was due to both practical and financial circumstances. The extremely rigorous inclusion criteria posed extensive restrictions on both PMD subjects and especially control recruitment. The substantial costs involved in repeated multiple tracer PET registrations also affected the sample size. However, the statistical effect of having a small sample size is that significant differences are difficult to detect: the statistical power might be too low, with the risk of imposing a type II error. Nonetheless, despite the small sample size, very strong statistical effects were demonstrated in our study. Statistically significant results in a study should be considered truly statistically significant and based on the significance level chosen, irrespective of the sample size, since the sample size is included in the testing. Another shortcoming of the study is that, for both practical and financial reasons, it had to be conducted in a stepwise fashion. Accordingly, scanners and software were replaced during the course of the study, resulting in the necessity for meticulous adaptations of the scanning and evaluation procedures, including correction for inter-scanner differences in radiotracer counts, to minimize the consequences of these replacements. However, the main finding of a completely different ratio of right-to-left dorsolateral prefrontal cortex serotonergic activation between the study groups is robust and could not even theoretically have been affected by the replacement of scanners or software during the study.

The use of SUV as an outcome measure has certain limitations, including a potential bias due to regional variations in blood flow and tracer clearance. Despite these limitations, SUV has been shown to correlate well with the net accumulation rate obtained by compartment modelling with a metabolite-corrected arterial input function, which is the gold standard method [31]. On analysing our ^15^O-H_2_O data, no significant corresponding group difference in dorsolateral prefrontal cortex radiotracer asymmetry was seen (unpublished data, not shown). In addition, the use of the SUV method allowed for repeated measurements within the same menstrual cycle, which would not have been feasible using arterial cannulation.

Another shortcoming of the study is that, when it was planned, MRI and computed tomography co-scan transformation support was not available, and thus all the ROIs had to be drawn without this support, inevitably reducing the anatomical precision. The calendar timing of our PET scans was not always optimal, but does reflect reality in clinical research settings. The counterbalanced study design, initially intended for the whole study and applied to the first cohort of eight PMD subjects, was theoretically a strength of the study, but did introduce unphysiological ‘split cycles’, which is why it was dismissed and only used when no logistic alternative was at hand. These drawbacks have to be taken into consideration when interpreting our data.

There are several methodological strengths of this study. The very strict inclusion and exclusion criteria of the study groups, resulting in a patient group with severe symptoms and a control group practically devoid of premenstrual symptoms; the similar ages of the group members; the external recruitment of controls, thus actively avoiding the otherwise so commonly used ‘convenience controls’; the use of identical acquisition protocols for PET data collection; the meticulously conducted PET scans at a standardized time of day; the standardized participant fasting state and body positioning; the standardized temperature, light and sound exposure; and the uniformity of blood sampling and sample handling are all methodological strengths of the study.

## Conclusion

It is well known that the dorsolateral prefrontal cortex is crucial for impulse control and for inhibition of affect and aggression [23, 74]. It is also known that, to a great extent, negative emotions are lateralized to the right hemisphere of the brain [66, 67]. The results of this study imply that women who do not experience premenstrual mood symptoms have an intrinsic means of increasing relative serotonin activity on the right side of the dorsolateral prefrontal cortex during the late luteal phase, possibly consequently dampening negative emotions that otherwise arise during this phase of sexual hormone decrease, and that women with severe premenstrual dysphoria either lack this mechanism completely or have weak, ineffective inhibition. This might explain why some women have no premenstrual mood symptoms while others suffer greatly. It might also explain the prompt, impressive effect of SSRIs on these symptoms. Thus, as was disclosed in this study, the cardinal symptom irritability is inversely related to the degree of increased relative serotonergic activity of the right dorsolateral prefrontal cortex in the premenstrual phase. Because of the small number of subjects in the study, the results should be considered preliminary, however, and should be verified in further larger studies.

## Supporting Information

S1 File Subject characteristics. (PDF)

S2 File Hormone data. (PDF)

S3 File Scanner correct SUV AUC values. (PDF)

S4 File VAS ratings for 14 variables. (PDF)

S5 File Metadata file. (PDF)
