# Supplementary material for: Women with Premenstrual Dysphoria Lack the Seemingly Normal Premenstrual Right-Sided Relative Dominance of 5-HTP-Derived Serotonergic Activity in the Dorsolateral Prefrontal Cortices - A Possible Cause of Disabling Mood Symptoms
Source: PLoS One. 2016 Sep 12;11(9):e0159538. doi: 10.1371/journal.pone.0159538 (PMC5019404; doi:10.1371/journal.pone.0159538)
Supplement: S1 File — (PDF) [file pone.0159538.s001.pdf]

NR	KAT	AGE	HEIGHT	WEIGHT	BMI	MADRS score	age at symptom start
1	1	34,8	171	63	21,5	4	28
1	1						
2	1	42,8	167	65	23,3	0	31
2	1						
3	1	38,7	169	59,7	20,9	1	30
3	1						
4	1	33,9	168	74,2	26,3	0	28,5
4	1						
5	1	40,7	167	62,3	22,3	3	20
5	1						
6	1	38,9	169	60,1	21	2	12
6	1						
7	1	35,9	161	56	21,6	MISSING	31
7	1						
8	1	45,7	167	63,5	22,8	4	35
8	1						
9	1	42,5	161	56,7	21,9	0	22,5
9	1						
10	1	39,5	169	58	20,3	0	19
10	1						
11	1	35,8	158,5	47,3	18,8	2	30
11	1						
12	1	37,6	164	60,8	22,6	2	23
12	1						
13	2	40,5	163	65,4	24,6	3	
13	2						
14	2	45,5	179,5	64,2	19,9	2	
14	2						
15	2	41,8	170	65,6	22,7	3	
15	2						
16	2	36,5	167	71,6	25,7	2	
16	2						
17	2	38	162	69,5	26,5	0	
17	2						
18	2	38,8	169	71,6	25,1	0	
18	2						
19	2	34,7	172	70	23,7	3	
19	2						
20	2	29,5	180	79	24,4	0	
20	2						

years of symptoms	number of symptoms	days with symptoms	number of pregnancies
6,8	7	10,5	3
11,8	11	8	0
8,7	6	9	6
5,4	8	7	3
20,7	5	7	0
26,9	9	14	2
4,9	5	7	3
10,7	6	14	2
20	11	14	3
20,5	8	8	3
5,8	10	3,5	4
14,6	9	10	6
			4
			3
			1
			4
			5
			5
			4
			3

number of children born	lenght of menstrual cycle	days of bleeding
3	28	6
0	28	4,5
3	28,5	7,5
3	26	5
0	27,5	6,5
2	26,5	5
2	28	5,5
1	28	5,5
2	31,5	6
1	28	5
1	27,5	5,6
3	32,5	6
4	25	5
3	25	5,5
2	27,5	5
4	30	7
2	26	4
3	28	5,5
3	29,5	5
2	25	3,5
