# Supplementary material for: Women with Premenstrual Dysphoria Lack the Seemingly Normal Premenstrual Right-Sided Relative Dominance of 5-HTP-Derived Serotonergic Activity in the Dorsolateral Prefrontal Cortices - A Possible Cause of Disabling Mood Symptoms
Source: PLoS One. 2016 Sep 12;11(9):e0159538. doi: 10.1371/journal.pone.0159538 (PMC5019404; doi:10.1371/journal.pone.0159538)
Supplement: S2 File — (PDF) [file pone.0159538.s002.pdf]

| NR | KAT | FAS | ESTRADIOL | PROGESTERONE | Cycle day of PET Foll |
|---|---|---|---|---|---|
| 1 | 1 | 1 | 151 | 1,18 | 7 |
| 1 | 1 | 2 | 168 | 23 | |
| 2 | 1 | 1 | 572 | 0,58 | 9 |
| 2 | 1 | 2 | 171 | 16 | |
| 3 | 1 | 1 | 265 | 8 | 14 |
| 3 | 1 | 2 | 163 | 10 | |
| 4 | 1 | 1 | 569 | 1,4 | 10 |
| 4 | 1 | 2 | 148 | 3,4 | |
| 5 | 1 | 1 | 512 | 1,38 | 11 |
| 5 | 1 | 2 | 266 | 15,8 | |
| 6 | 1 | 1 | 168 | 2,4 | 6 |
| 6 | 1 | 2 | 453 | 45 | |
| 7 | 1 | 1 | 213 | 1,32 | 9 |
| 7 | 1 | 2 | 260 | 14,1 | |
| 8 | 1 | 1 | 385 | 1,46 | 7 |
| 8 | 1 | 2 | 456 | 78 | |
| 9 | 1 | 1 | 204 | 2,6 | 7 |
| 9 | 1 | 2 | 407 | 92 | |
| 10 | 1 | 1 | 1699 | 1,21 | 10 |
| 10 | 1 | 2 | 169 | 3,7 | |
| 11 | 1 | 1 | 177 | 1,99 | 10 |
| 11 | 1 | 2 | 874 | 48 | |
| 12 | 1 | 1 | 370 | 1,67 | 7 |
| 12 | 1 | 2 | 192 | 11,4 | |
| 13 | 2 | 1 | 2709 | 1,63 | 10 |
| 13 | 2 | 2 | 561 | 36 | |
| 14 | 2 | 1 | 265 | 1,35 | 9 |
| 14 | 2 | 2 | 453 | 34 | |
| 15 | 2 | 1 | 350 | 0,9 | 8 |
| 15 | 2 | 2 | 209 | 7,7 | |
| 16 | 2 | 1 | 302 | 1,37 | 12 |
| 16 | 2 | 2 | 326 | 21 | |
| 17 | 2 | 1 | 325 | 1,43 | 11 |
| 17 | 2 | 2 | 332 | 29 | |
| 18 | 2 | 1 | 445 | 3,4 | 12 |
| 18 | 2 | 2 | 287 | 26 | |
| 19 | 2 | 1 | 267 | 1,13 | 9 |
| 19 | 2 | 2 | 101 | 2,6 | |
| 20 | 2 | 1 | 217 | 0,58 | 10 |
| 20 | 2 | 2 | 1061 | 8,3 | |

| Cycle day of PET Lut | Natural menstrual cycle | Reversed menstrual cycle |
|---|---|---|
| -5 | | 1 |
| -3 | 1 | |
| -1 | | 1 |
| -1 | | 1 |
| -3 | | 1 |
| -6 | 1 | |
| -4 | 1 | |
| -8 | | 1 |
| -7 | 1 | |
| -1 | 1 | |
| -3 | 1 | |
| -2 | | 1 |
| -3 | 1 | |
| -5 | 1 | |
| -1 | 1 | |
| -8 | 1 | |
| -5 | 1 | |
| -2 | 1 | |
| -1 | | 1 |
| -8 | 1 | |
