# Supplementary material for: Women with Premenstrual Dysphoria Lack the Seemingly Normal Premenstrual Right-Sided Relative Dominance of 5-HTP-Derived Serotonergic Activity in the Dorsolateral Prefrontal Cortices - A Possible Cause of Disabling Mood Symptoms
Source: PLoS One. 2016 Sep 12;11(9):e0159538. doi: 10.1371/journal.pone.0159538 (PMC5019404; doi:10.1371/journal.pone.0159538)
Supplement: S3 File — (PDF) [file pone.0159538.s003.pdf]

The SAS System

Obs	NR	FAS	AGE	KAT	AUCWHB	AUCNCDX	AUCNCSIN	AUCPUTDX	AUCPUTSIN	AUCPFCDX	AUCPFCSIN	AUCMFCDX	AUCMFCSIN
1	1	1	34.8	1	61.1925	72.6775	66.4800	83.725	83.725	57.0725	59.1450	60.7450	59.6525
2	1	2	34.8	1	61.1275	71.4400	72.4350	80.763	86.475	57.7575	62.1300	63.4050	62.6825
3	2	1	42.8	1	60.7025	65.4825	75.4725	76.515	80.668	59.8575	61.0125	61.4575	61.9350
4	2	2	42.8	1	54.0475	56.8825	65.7575	74.895	74.378	55.9350	56.0950	56.0275	54.3850
5	3	1	38.7	1	55.8475	75.5325	75.4175	81.123	79.993	58.3950	60.4125	61.4550	63.2250
6	3	2	38.7	1	60.6700	79.5475	79.1800	84.635	83.645	59.9825	62.1450	66.6925	67.4075
7	4	1	33.9	1	69.3400	84.5075	88.1100	98.165	98.170	71.3825	73.2750	75.4175	74.6450
8	4	2	33.9	1	62.8550	76.9450	78.3500	88.970	93.445	64.5750	65.5250	67.3225	66.2025
9	5	1	40.7	1	53.8300	68.3025	64.6100	71.045	75.803	53.2975	52.7100	53.3650	55.6050
10	5	2	40.7	1	51.5275	64.4375	64.5275	71.573	64.528	50.7975	50.4550	53.8900	55.5800
11	6	1	38.9	1	67.9825	79.4075	80.9900	89.633	88.648	61.8050	63.9950	62.7175	66.9925
12	6	2	38.9	1	57.8250	65.1650	67.0725	70.458	69.893	49.5750	52.6550	57.4675	59.7925
13	7	1	35.9	1	65.9575	80.4950	79.8925	92.385	89.758	65.1375	66.8725	68.9200	68.9925
14	7	2	35.9	1	64.9275	75.7775	82.0500	82.290	90.495	64.1225	65.8775	67.6675	68.1050
15	8	1	45.7	1	54.2800	71.9900	67.5475	72.495	76.783	55.6975	56.4600	59.5575	55.3675
16	8	2	45.7	1	57.9950	73.1825	77.2275	76.380	78.123	57.0375	57.9000	61.5375	61.8775
17	9	1	42.5	1	55.7634	66.5746	61.5290	78.388	74.345	58.6819	54.9423	54.0082	59.1698
18	9	2	42.5	1	57.5395	78.4686	76.9127	79.197	83.877	57.6496	56.6083	51.1819	55.1922
19	10	1	39.5	1	51.6371	64.1380	65.1347	69.199	67.176	52.5296	53.0324	44.0597	47.3888
20	10	2	39.5	1	51.3336	56.0252	58.7325	65.075	68.598	53.1127	52.2142	51.6906	52.4165
21	11	1	35.8	1	50.9796	65.5541	65.7653	68.895	64.653	49.3077	48.5847	52.7408	50.5274
22	11	2	35.8	1	54.8471	65.0662	65.4530	68.615	70.897	54.4217	54.0825	57.3193	59.8987
23	12	1	37.6	1	62.7993	76.3385	64.3403	78.240	75.410	59.8183	59.3304	67.3570	64.3046
24	12	2	37.6	1	57.4347	68.9608	56.4485	69.178	72.957	54.5226	55.3953	53.9060	58.8087
25	13	1	40.5	2	70.3885	86.6796	85.2427	90.015	87.218	65.7713	69.6210	68.8683	71.8254
26	13	2	40.5	2	67.3094	81.7530	81.0212	89.562	87.614	62.7368	61.8949	67.8925	69.0974

The SAS System

Obs	NR	FAS	AGE	KAT	AUCWHB	AUCNCDX	AUCNCSIN	AUCPUTDX	AUCPUTSIN	AUCPFCDX	AUCPFCSIN	AUCMFCDX	AUCMFCSIN
**27**	14	1	45.5	2	51.0004	61.1928	57.0248	71.471	68.571	52.0476	53.5738	53.1662	51.7353
**28**	14	2	45.5	2	50.0336	59.5833	52.5802	64.007	72.763	51.2712	51.0332	53.1514	51.0867
**29**	15	1	41.8	2	50.7981	61.8740	61.5557	68.303	70.347	49.5903	50.5810	50.0306	53.8326
**30**	15	2	41.8	2	48.7722	65.7921	53.7226	64.444	68.595	48.1534	47.9064	48.3705	46.5617
**31**	16	1	36.5	2	74.1578	84.1806	80.5928	93.302	96.066	72.4056	71.4298	73.6253	77.5880
**32**	16	2	36.5	2	79.3938	96.1937	83.6957	102.159	95.896	80.6463	74.3929	83.1423	78.5013
**33**	17	1	38	2	58.0988	68.8921	61.8354	73.066	74.765	53.9606	56.8552	57.6615	61.6063
**34**	17	2	38	2	58.5242	64.0369	61.6331	79.415	71.207	54.7370	55.1446	56.1799	60.3776
**35**	18	1	38.8	2	51.6851	69.7345	62.7558	67.353	69.161	51.1146	52.0620	55.8415	56.0184
**36**	18	2	38.8	2	49.8679	57.6305	56.8771	58.874	70.708	52.2104	51.2742	54.3914	49.6501
**37**	19	1	34.7	2	58.4852	67.0297	66.9948	79.648	77.970	63.1585	61.5304	60.7927	60.8613
**38**	19	2	34.7	2	64.4745	90.1838	86.4625	90.279	96.333	70.5131	68.5192	68.4011	68.3283
**39**	20	1	29.5	2	74.7957	92.4805	92.7722	104.835	101.343	71.9216	73.8084	70.7250	73.4240
**40**	20	2	29.5	2	70.1700	80.0139	84.8582	92.298	93.409	69.9907	68.0260	68.6935	70.8260
