# Supplementary material for: Women with Premenstrual Dysphoria Lack the Seemingly Normal Premenstrual Right-Sided Relative Dominance of 5-HTP-Derived Serotonergic Activity in the Dorsolateral Prefrontal Cortices - A Possible Cause of Disabling Mood Symptoms
Source: PLoS One. 2016 Sep 12;11(9):e0159538. doi: 10.1371/journal.pone.0159538 (PMC5019404; doi:10.1371/journal.pone.0159538)
Supplement: S4 File — (PDF) [file pone.0159538.s004.pdf]

The SAS System

Obs	NR	KAT	FAS	HUV	SVULL	GLAD	SP_ND	LUGN	SEXBE	UNDSM	BEHS_T	V_NL	ENERG	IRRIT	TR_TT	NEDST	BR_STSP
1	1	1	1	0	0	48	49	24	12	0	0	89	0	0	100	50	0
2	1	1	2	100	11	19	49	48	1	0	25	38	0	70	70	39	0
3	2	1	1	0	1	92	0	94	50	1	1	88	88	0	1	0	0
4	2	1	2	75	76	20	8	83	15	0	71	12	13	81	12	89	0
5	3	1	1	1	2	100	0	100	99	3	0	100	100	0	0	0	0
6	3	1	2	10	40	45	45	47	26	26	33	46	93	50	4	47	47
7	4	1	1	20	1	98	1	99	12	17	2	98	98	1	6	1	0
8	4	1	2	72	22	0	52	1	1	54	0	2	1	99	77	73	100
9	5	1	1	28	28	100	0	100	100	0	0	100	48	0	49	0	0
10	5	1	2	0	30	68	18	77	100	1	1	67	42	82	51	63	29
11	6	1	1	2	2	98	2	2	56	2	1	98	97	3	2	2	2
12	6	1	2	18	3	3	60	25	8	2	2	10	8	100	82	98	1
13	7	1	1	11	0	83	12	87	1	0	0	100	0	0	0	0	0
14	7	1	2	0	61	14	78	12	0	0	0	8	0	67	1	15	0
15	8	1	1	0	0	68	18	48	0	0	0	52	55	0	0	1	0
16	8	1	2	0	15	56	68	43	0	21	0	62	57	51	32	39	0
17	9	1	1	2	8	73	3	83	26	12	5	83	68	2	48	11	1
18	9	1	2	3	47	15	25	3	3	90	79	2	3	32	96	96	95
19	10	1	1	13	1	89	4	92	45	0	1	90	89	17	3	10	0
20	10	1	2	69	47	35	22	56	0	25	19	45	34	47	47	48	0
21	11	1	1	0	0	100	0	91	86	0	0	87	85	0	0	0	0
22	11	1	2	70	59	7	9	15	82	31	63	18	11	78	61	69	30
23	12	1	1	6	2	97	2	95	93	1	3	97	77	2	3	3	3
24	12	1	2	8	33	45	40	27	71	25	80	75	43	38	33	40	53
25	13	2	1	2	2	80	1	89	11	3	3	91	6	5	36	11	3
26	13	2	2	2	2	89	12	85	3	2	16	77	80	5	8	3	54

The SAS System

Obs	NR	KAT	FAS	HUV	SVULL	GLAD	SP_ND	LUGN	SEXBE	UNDSM	BEHS_T	V_NL	ENERG	IRRIT	TR_TT	NEDST	BR_STSP
**27**	14	2	1	0	0	80	22	54	9	1	0	73	64	3	26	1	0
**28**	14	2	2	0	0	76	4	86	29	0	0	73	44	4	10	0	0
**29**	15	2	1	4	4	62	6	64	6	6	6	65	45	4	4	4	4
**30**	15	2	2	5	24	65	8	70	4	4	5	61	61	4	14	4	36
**31**	16	2	1	6	0	92	0	94	10	0	11	90	30	0	12	0	0
**32**	16	2	2	0	0	89	0	89	0	0	7	88	38	0	0	0	0
**33**	17	2	1	18	2	59	36	47	6	2	42	76	57	15	28	9	1
**34**	17	2	2	0	9	88	23	54	14	18	46	73	61	9	23	14	1
**35**	18	2	1	2	3	67	7	83	17	1	9	63	58	3	27	1	1
**36**	18	2	2	1	2	64	3	85	3	1	36	57	41	14	58	1	1
**37**	19	2	1	2	8	27	37	18	31	2	35	42	44	14	18	26	1
**38**	19	2	2	1	1	46	12	41	23	2	14	33	27	2	20	1	0
**39**	20	2	1	0	0	99	13	99	2	1	1	98	83	1	22	0	1
**40**	20	2	2	1	16	100	2	99	22	0	1	96	98	2	4	0	0
