# Supplementary material for: Women with Premenstrual Dysphoria Lack the Seemingly Normal Premenstrual Right-Sided Relative Dominance of 5-HTP-Derived Serotonergic Activity in the Dorsolateral Prefrontal Cortices - A Possible Cause of Disabling Mood Symptoms
Source: PLoS One. 2016 Sep 12;11(9):e0159538. doi: 10.1371/journal.pone.0159538 (PMC5019404; doi:10.1371/journal.pone.0159538)
Supplement: S5 File — (PDF) [file pone.0159538.s005.pdf]

Metadata file – defining the variables in the three supplementary data files:

1. Subject characteristics
2. Hormone data
3. Scanner corrected SUV AUC values
4. VAS ratings for 14 variables

Subject characteristics

Rows 1-40 = observations

Column NR = subject number

Column FAS = menstrual phase (1 = follicular, 2 = premenstrual)

Column KAT = subject category (1 = PMD, 2 = control)

Column MADRS score = score at MADRS self-test at study entry

Column 'age at symptom start' = age at start of PMD symptoms

Column 'years of symptoms' = number of years of PMD

Column 'number of symptoms' = number of experienced PMD symptoms per menstrual cycle

Column 'days of symptoms' = number of days of PMD symptoms per menstrual cycle

Column 'days of bleeding' = number of menstrual days per menstrual cycle

Hormone data

Rows 1-40 = observations

Column NR = subject number

Column KAT = subject category (1 = PMD, 2 = control)

Column FAS = menstrual phase (1 = follicular, 2 = premenstrual)

Column ESTRADIOL = plasma level of estradiol (pmol/L)

Column PROGESTERONE = plasma level of progesterone (nmol/L)

Column Cycle day of PET Foll = the menstrual cycle day when follicular phase PET was done (counted from the first day of menstrual bleeding)

Column Cycle day of PET Lut = the menstrual cycle day when premenstrual phase PET was done (counted from the first day of menstrual bleeding)

Column Natural menstrual cycle = both PET registrations done in the same menstrual cycle

Column Reversed menstrual cycle = Premenstrual phase PET was done before Follicular phase PET (= in two different menstrual cycles)

Scanner corrected SUV AUC values:

Rows 1-40 = observations

Column NR = Subject number

Column FAS = menstrual phase (1 = follicular, 2 = premenstrual)

Column AGE = age of subject at PET scan

Column KAT = subject category (1 = PMD, 2 = control)

Column AUCWHB = AUC of SUV for the whole brain ROI

Column AUCNCDX = AUC of SUV for the right caudate nucleus ROI

Column AUCNCSIN = AUC of SUV for the left caudate nucleus ROI

Column AUCPUTDX = AUC of SUV for the right putamen ROI

Column AUCPUTSIN = AUC of SUV for the left putamen ROI

Column AUCPFCDX = AUC of SUV for the right dorsolateral prefrontal cortex ROI

Column AUCPFCSIN = AUC of SUV for the left dorsolateral prefrontal cortex ROI

Column AUCMFCDX = AUC of SUV for the right mediofrontal cortex ROI

Column AUCMFCSIN = AUC of SUV for the left mediofrontal cortex ROI

VAS ratings for 14 variables:

Rows 1-40 = observations

Column NR = subject number

Column KAT = subject category (1 = PMD, 2 = control)

Column FAS = menstrual phase (1 = follicular, 2 = premenstrual)

Column HUV = VAS rating for the variable 'headache'

Column SVULL = VAS rating for the variable 'bloating'

Column GLAD = VAS rating for the variable 'happiness'

Column SP_ND = VAS rating for the variable 'tension'

Column LUGN = VAS rating for the variable 'relaxation'

Column SEXBE = VAS rating for the variable 'sexual desire'

Column UNDSM = VAS rating for the variable 'pelvic pain'

Column BEHS_T = VAS rating for the variable 'craving for sweets'

Column V_NL = VAS rating for the variable 'friendliness'

Column ENERG = VAS rating for the variable 'energy'

Column IRRIT = VAS rating for the variable 'irritability'

Column TR_TT = VAS rating for the variable 'fatigue'

Column NEDST = VAS rating for the variable 'depressed mood'

Column BR_STSP = VAS rating for the variable 'breast tenderness'
